# Improving Mild to Moderate Depression With an App-Based Self-Guided Intervention: Protocol for a Randomized Controlled Trial

**DOI:** 10.2196/46651

**Published:** 2023-10-25

**Authors:** Ina Beintner, André Kerber, Clara Dominke, Ulrich Voderholzer

**Affiliations:** 1 MindDoc Health GmbH Munich Germany; 2 Division of Clinical–Psychological Intervention Department of Education and Psychology Freie Universität Berlin Berlin Germany; 3 Schoen Clinic Roseneck Prien am Chiemsee Germany; 4 Department of Psychiatry and Psychotherapy University Hospital Ludwig Maximilian University of Munich Munich Germany; 5 Department of Psychiatry and Psychotherapy Faculty of Medicine, Medical Center University of Freiburg Freiburg Germany

**Keywords:** depression, mobile app, intervention, unguided, transdiagnostic, randomized controlled trial, e-mental health, digital app, self-management, mental health, mHealth, mobile health, unguided digital intervention, public health, digital intervention, mobile phone

## Abstract

**Background:**

Depression is one of the most prevalent mental disorders and frequently co-occurs with other mental disorders. Despite the high direct and indirect costs to both individuals and society, more than 80% of those diagnosed with depression remain with their primary care physician and do not receive specialized treatment. Self-guided digital interventions have been shown to improve depression and, due to their scalability, have a large potential public health impact. Current digital interventions often focus on specific disorders, while recent research suggests that transdiagnostic approaches are more suitable.

**Objective:**

This paper presents the protocol for a study that aims to assess the efficacy of a self-guided transdiagnostic app-based self-management intervention in patients with mild or moderate depression with and without comorbid mental disorders. Specifically, we are investigating the impact of the intervention on symptoms of depression, quality of life, anxiety symptoms, and mental health–related patient empowerment and self-management skills.

**Methods:**

The intervention under investigation, MindDoc with Prescription, is a self-guided digital intervention aimed at supporting individuals with mild to moderate mental disorders from the internalizing spectrum, including depression. The app can be used as a low-threshold psychosocial intervention. Up to 570 adult patients will be randomized to either receive the intervention in addition to care as usual or only care as usual. We are including adults with a permanent residency in Germany and mild or moderate depression according to International Classification of Diseases, 10th Revision, criteria (F32.0, F32.1, F33.0, and F33.1). Clinical interviews will be conducted to confirm the diagnosis. Data will be collected at baseline as well as 8 weeks and 6 months after randomization. The primary outcome will be depression symptom severity after 8 weeks. Secondary outcomes will be quality of life, anxiety symptom severity, and patient empowerment and self-management behaviors. Data will be analyzed using multiple imputations, using the intention-to-treat principle, while sensitivity analyses will be based on additional imputation strategies and a per-protocol analysis.

**Results:**

Recruitment for the trial started on February 7, 2023, and the first participant was randomized on February 14, 2023. As of September 5, 2023, 275 participants have been included in the trial and 176 have provided the primary outcome. The rate of missing values in the primary outcome is approximately 20%.

**Conclusions:**

Data from this efficacy trial will be used to establish whether access to the intervention is associated with an improvement in depression symptoms in individuals diagnosed with mild or moderate depression. The study will contribute to expanding the evidence base on transdiagnostic digital interventions.

**Trial Registration:**

German Registry of Clinical Trials DRKS00030852; https://drks.de/search/de/trial/DRKS00030852

**International Registered Report Identifier (IRRID):**

DERR1-10.2196/46651

## Introduction

In Germany, approximately 1 in 10 individuals experience depression in a given 12-month period [1]. Depression frequently co-occurs with other mental disorders; epidemiological data show that 61% of those with depression have at least 1 other mental illness [2]. This is supported by clinical evidence, where 69% of patients diagnosed with major depression also had at least 1 other Axis I diagnosis [3].

Health insurance billing data in Germany show a comorbidity rate of 50% within the group of mental disorders [4]. The most common combinations involve mood disorders (F3 group) and neurotic, stress, and somatoform disorders (F4 group) according to the International Classification of Diseases, 10th Revision (ICD-10), classification system [5].

The World Health Organization’s World Mental Health Survey findings suggest that the majority of comorbid mental illnesses are a result of shared causal factors [6]. A study of the Dunedin Multidisciplinary Health and Development Study data also supports this conclusion [7], showing that across diagnostic categories mental illness can be broken down into 3 common higher-level factors (internalizing, externalizing, and thought disorders).

This finding may explain the transdiagnostic effects of many pharmaceutical and psychosocial treatments for a range of mental disorders [8,9]. As a result, mental health treatment approaches are shifting toward transdiagnostic interventions which are based on common underlying mechanisms of mental illness that have been proven to be effective across diagnoses [10]. Examples include the Unified Protocol [11] and the Common Elements Treatment Approach [12], which are increasingly replacing disorder-specific treatments.

Mental disorders are among the top 4 causes of healthy life years lost in Germany, with depression taking the first place within that group [13]. In 2015, the direct medical costs of depression in Germany were estimated at nearly €9 billion (US $8.4 billion) [14], while the loss of income due to depression throughout the European Union is approximately €176 billion (US $164 billion) per year [15].

Despite the high prevalence of depression and its significant direct and indirect costs, few receive specialized treatment. Health insurance billing data showed that while nearly 10% of insured individuals were diagnosed with an affective disorder at least once over a 3-year period, more than 80% of those affected remained with their primary care physician for the entire treatment [4]. On top, epidemiological data suggest that 3 out of 4 cases of major depression in Germany go undiagnosed and only 1 in 10 affected individuals receive evidence-based treatment as per national guidelines [16]. More recent data confirm this trend [17].

Treatment delay for depression can be attributed not only to limited resources among providers, but also to personal barriers such as low health literacy or negative attitudes toward seeking help. A desire to handle the issue alone and a low perceived need for treatment play a greater role in delaying treatment than structural barriers [18,19]. These factors hinder early detection, worsen the issue of underdiagnosis, and increase the risk of long-term symptom deterioration and chronicity [20].

Most people with depression in Germany receive treatment only from primary care physicians [4]. However, billing data from 2021 indicate that there are significant discrepancies between the frequency of mental illnesses in the general population as detected in epidemiological studies, and the diagnoses made by general practitioners [1,21,22]. While depression is diagnosed relatively in line with its frequency in the general population, anxiety disorders are underdiagnosed by as much as 75%. This suggests that comorbid disorders are likely not consistently recognized in primary care. This may be due to the difficulty of making a guideline-based diagnosis and differential diagnosis within the limited time frame of a general practitioner consultation, which typically lasts between 6 and 11 minutes [23,24].

Studies have found digital, cognitive-behavioral self-management programs to be effective for depression. Disorder-specific programs produce moderate to large effects on depression compared to waitlist controls [25-30]. Specifically, smartphone-based interventions have been shown to produce small to moderate effects [31]. The effect of self-guided transdiagnostic interventions on depression symptoms is comparable to the effect of disorder-specific interventions, with moderate to large effects on depression symptoms, and large effects on anxiety symptoms [32].

Interventions that include guidance by a mental health professional have larger effects [30,33-36], but there is also research showing no differences between guided and self-guided interventions [34,37-39]. Overall, self-guided interventions are more scalable due to lower costs and less human resources, making them more impactful for public health [40], especially when administered in primary care settings [41]. Other factors contributing to the variability in the results of self-guided interventions include study design characteristics including the use of clinical interviews [42], automated reminders [43], and the clinical setting [41], as well as participant characteristics like the severity of the disorder [34].

Meta-analyses confirm the effectiveness of digital interventions not only in reducing symptoms but also in improving quality of life [44] and there is also some evidence that such interventions can reduce self-stigma [45-47] and improve health-promoting behaviors [48].

Overall, digital interventions can help close the treatment gap for mental disorders including depression. In primary care settings, low-threshold transdiagnostic interventions appear to be most suitable, particularly due to the opportunity to address comorbid disorders, which are often overlooked in the setting, with the same intervention.

In a previous study on such a transdiagnostic intervention (MindDoc App) in people with mild or moderate symptoms of mental disorders from the internalizing spectrum [49,50], we detected small effects on depression symptoms (*d*=0.34) in both the overall sample (*d*=0.34) and a subsample of participants with depression symptoms above the diagnostic threshold (*d*=0.43). Small effects were also detected on quality of life (overall sample: *d*=0.19; subsample: *d*=0.31), anxiety symptoms (overall sample: *d*=0.22; subsample: *d*=0.24), and mental health–related patient empowerment and self-management skills (overall sample: *d*=0.29; subsample: *d*=0.41). Patient empowerment and self-management behaviors encompass a range of attitudes and behaviors that individuals exhibit in relation to taking control of their health and well-being, including seeking support, actively participating in health care decision-making, and adopting healthy habits.

The aim of this study is to evaluate the intervention in a sample of individuals diagnosed with mild or moderate depression. Based on our previous findings, we expect the intervention to be associated with less severe symptoms of depression at the end of an 8-week intervention period compared with a waitlist control group. We will also examine intervention effects after 6 months, as well as intervention effects on quality of life, anxiety symptoms, and mental health–related patient empowerment and self-management skills.

## Methods

### Study Design

**Figure 1:**
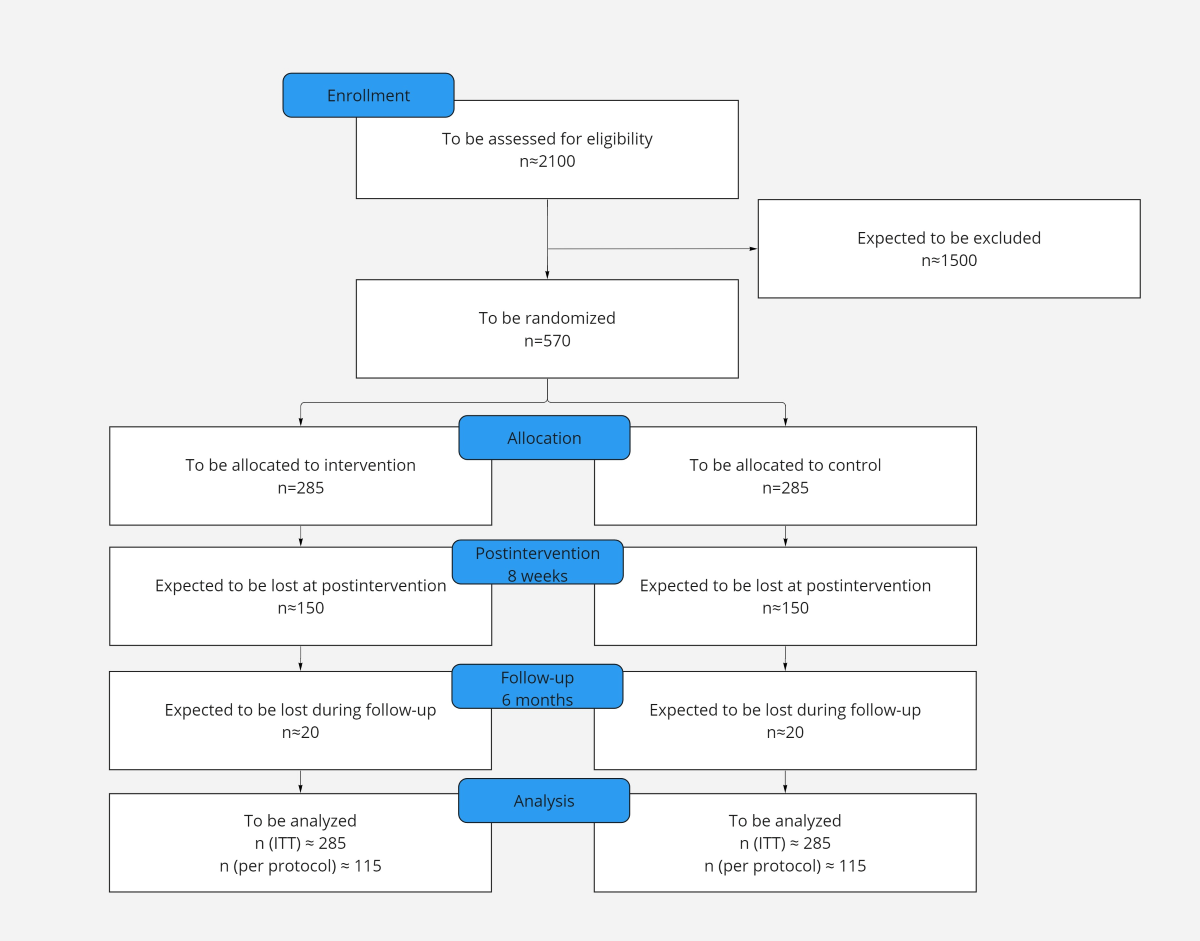
Study design. ITT: intention-to-treat.

We are conducting a monocenter 2-arm randomized controlled trial with 3 assessment points (baseline, postintervention at 8 weeks, and follow-up at 6 months). Participants in the intervention condition will receive access to MindDoc with Prescription in addition to care as usual. Participants in the control condition will only have access to care as usual. Care as usual includes any form of treatment, including psychotherapy, unless psychotherapy has already been started or planned at the time of inclusion in the study. The study design is illustrated in Figure 1.

### Procedures

Study participation includes a web-based screening, a diagnostic interview (baseline), 3 additional web-based surveys (baseline, postintervention, and follow-up) and a clinician rating (postintervention). In the intervention group, it additionally includes access to a digital health application (DiGA). The process is described in more detail below. All study data will be collected in an electronic data capture (EDC) system provided by Climedo Health GmbH in the form of electronic case report forms and electronic patient reported outcome forms.

Applicants first receive written information on the study procedure and must explicitly agree to participate in the study via electronic consent by ticking a number of checkboxes before they can proceed to the screening questionnaire. The responses are logged in the study database and can only be changed by the participants themselves. By ticking the checkboxes, they confirmed that they had thoroughly read and understood the information about the study procedures and data security measures (including information on how to contact the study team if they had questions) provided to them. This approach is General Data Protection Regulation (GDPR) compliant and was approved by the ethics committee.

After consent has been given, applicants proceed to a screening questionnaire, on which the majority of exclusion criteria are assessed. The applicants who do not fulfill any exclusion criteria are then invited to the diagnostic interview.

The diagnostic interview is conducted via telephone by a clinical psychologist or clinical psychologist in training. Before the actual interview begins, the participants are given another opportunity to ask questions about the study. They are also informed verbally once again that their participation is voluntary and that they can withdraw their consent at any time without giving reasons and without disadvantages. Immediately after the interview, applicants receive feedback on whether or not they can participate in the study.

The diagnostic interview is then followed by the actual baseline survey for the study. The link to the survey is sent to the participant’s email address.

After the baseline survey has been completed, participants are randomized at the person level with 1:1 group assignment. Randomization is performed as block randomization in blocks of 4, 6, and 8, stratified by sex (male, female, and diverse), diagnosis (F32.0, F32.1, F33.0, and F33.1), and previous psychiatric or psychotherapeutic treatment (yes and no). Randomization lists were generated (1 per stratum) using Sealed Envelope [51] and directly integrated into the EDC system. Randomization is performed in a protected area of the EDC system that can only be accessed by authorized members of the study team without them having access to the randomization lists.

Participants will be notified of the results of the assignment via email. Participants assigned to the intervention group will immediately receive free access to the DiGA described below. Participants in the control group will receive free access to the DiGA after they complete their follow-up assessment.

In both groups, postintervention and follow-up assessments will take place after 8 weeks and after 6 months. The links to the respective surveys are sent to the participant’s email address. The postintervention assessment also includes another telephone interview which is conducted by a clinical psychologist or clinical psychologist in training. Interviewers are blinded to the group assignment of the study participants.

If a participant fails to respond to a survey invitation, up to 5 reminders are sent within 14 days. If no response has been received after 28 days, the assessment will be marked as missing.

### Eligibility Criteria

#### Overview

Eligibility criteria were selected to ensure an optimal fit of the study sample with respect to the subsequent implementation of the app in the German health care setting, to meet the requirements of the legal authorities, to minimize the impact of confounding variables, and to ensure comparability with other studies on self-guided digital interventions.

#### Inclusion Criteria

We are including adults with mild or moderate depression according to ICD-10 criteria (F32.0, F32.1, F33.0, and F33.1). Clinical interviews are conducted to confirm the diagnosis. Raters are clinical psychologists and clinical psychologists in training who are not part of the core study team. They administer the short version of the diagnostic interview for mental disorders [52], a short structured clinical interview for diagnosing mental disorders. The interview assesses current and lifetime diagnoses according to the Diagnostic and Statistical Manual of Mental Disorders, Fifth Edition (DSM-5) and ICD-10.

Participants must also be fully legally competent, reside in Germany, and have sufficient German language skills. They must have access to their own smartphone (Android, version 6.0, Google, or iOS, version 13.0 or higher, Apple) and the internet, and be willing and able to use the app regularly and participate in the assessments.

#### Exclusion Criteria

We are excluding individuals who receive outpatient psychotherapy or inpatient treatment for a mental disorder at the point of enrollment or already have such a treatment planned to start within the next 8 weeks. We are also excluding individuals who report changes in antidepressant medication in the past 4 weeks, who report suicidal ideations, or who have a history of bipolar disorder, psychotic disorder, substance use disorder, or suspected substance use disorder. Individuals who report having used the MindDoc App or a comparable digital intervention in the past 12 months will also be excluded.

### Recruitment

Participants are recruited via press releases, social media advertising, and cooperating primary care practices. Recruitment will take place over a period of at least 6 months. It is expected that about 2500 prospective participants will have to be screened, of whom 800 will be invited for a diagnostic interview to determine eligibility.

### Intervention

MindDoc with Prescription is a digital intervention aimed at supporting individuals with mild to moderate mental disorders from the internalizing spectrum, including depression. The app can be used as a low-threshold psychosocial intervention.

MindDoc with Prescription is a risk class I medical device according to Annex VIII, Rule 11 of the Medical Device Regulation (REGULATION (EU) 2017/745 on medical devices). Intended users include individuals with mild and moderate mental disorders.

The app consists of four key components: (1) continuous self-monitoring of symptoms and personal resources (journal), (2) personalized feedback on symptoms and coping strategies (insights), (3) regular overall assessments of emotional health (result), and (4) courses and exercises for psychoeducation and self-management (discover). All content was developed by licensed clinical psychologists based on established guidelines for the treatment of mental disorders.

The journal component allows users to log symptoms and personal information up to 3 times a day, with mood assessments and free text inputs. The insights section provides personalized feedback based on the information recorded in the journal. The result section provides an overall assessment of emotional health every 14 days, while the discover section offers courses and exercises on coping with symptoms, drawing on cognitive behavioral therapy, and other evidence-based treatments. Recommendations for relevant courses are provided based on users’ journal entries. The app is designed to help patients understand and manage their symptoms, promoting self-sufficiency, and empowering them to take control of their mental health.

Participants can contact the study team and the MindDoc customer support by email at any time with questions about app use. Requests will typically be answered within 1 working day.

A detailed description of MindDoc with Prescription is provided in Multimedia Appendix 1. Instructions for use can be viewed on the app manufacturer’s website [53].

### Measures

#### Screening and Diagnostic Interview

The screening questionnaire is designed to assess the inclusion and exclusion criteria described above and includes the depression module of the Patient Health Questionnaire-9 (PHQ-9) [54,55] to assess depression symptom severity, the Alcohol Use Disorders Identification Test (AUDIT) [56] to assess problematic alcohol use, and the Drug Use Disorders Identification Test (DUDIT) [57] to assess drug use. It also contains questions on current and previous treatments and diagnoses, as well as questions on sociodemographic data.

The diagnostic interview is a shortened version of the short version of the diagnostic interview for mental disorders (Mini-DIPS) [52], a short structured clinical interview for diagnosing mental disorders. The interview assesses current and lifetime diagnoses according to DSM-5 and ICD-10 and is used to confirm that participants fulfill the diagnostic criteria for depression and to assess comorbidity.

#### Study End Points

##### Depression Symptom Severity

Depression symptom severity is assessed using the depression module of the PHQ-9 [53,58], a 9-item questionnaire that is part of the Primary Care Evaluation of Mental Disorders (PRIME-MD) diagnostic instrument for common mental disorders [54] and can be self-administered. The questionnaire evaluates each of the 9 DSM-5 diagnostic criteria for depression [59], with scores ranging from 0 (not at all) to 3 (nearly every day). The PHQ-9 is a reliable and valid tool for measuring depression, with a Cronbach α coefficient of .89.

##### Quality of Life

Quality of life is assessed with the Assessment of Quality of Life-8 Dimensions (AQoL-8D) [60]. The 35-item self-rating scale can be used to evaluate the impact of health care services on an individual’s psychosocial quality of life. It measures 6 psychosocial functioning domains and physical autonomy, and has shown high reliability (with a Cronbach α score of .96) and convergent and predictive validity.

##### Anxiety Symptom Severity

Anxiety symptom severity is assessed with the Generalized Anxiety Disorder-7 (GAD-7) [58,61], which is a 7-item tool for detecting symptoms of generalized anxiety disorder as defined in the DSM-5. Each item is rated on a scale from 0 (not at all) to 3 (nearly every day), making it an effective and reliable way to screen for anxiety disorders and assess their severity in both clinical settings and research.

##### Patient Empowerment and Self-Management Behaviors

Patient empowerment and self-management behaviors are assessed with a questionnaire we have developed for our previous study [49,50]. It consists of 10 statement items assessing patient empowerment on a 6-point Likert scale (strongly agree, mostly agree, somewhat agree, somewhat disagree, mostly disagree, and strongly disagree) and 18 question items assessing the frequency of self-management behaviors in the past 8 weeks on a 6-point Likert scale (never, once in a while, less than half the time, more than half the time, most of the time, and all the time). The questionnaire has good internal consistency in data from a previously conducted study. The parallel analysis yields 2 components, and an exploratory factor analysis with 2 factors yields medium to high factor loadings of the respective items on their scales as well as low cross-loadings. Reliabilities (McDonalds ω) are in the good to very good range for both subscales as well as the total scale (ω (self-management)=0.87; ω (empowerment)=0.82; ω (total scale)=0.89). Furthermore, the overall scale of the instrument showed convergent validity in the form of significant correlations (*P*<.01) with further surveyed constructs from health literacy (knowledge of self-help measures: *r*=0.44, 95% CI 0.39-0.49; “first aid skills”: *r*=0.38, 95% CI 0.33-0.43), with attitudes toward mental illness (positive attitudes toward help-seeking behavior: *r*=0.46, 95% CI 0.41-0.51; stigma toward mental illness: *r*=0.30, 95% CI 0.25-0.36), and with the “coping” (*r*=0.40, 95% CI 0.35-0.45) and “relationships” (*r*=0.37, 95% CI 0.31-0.42) subscales of the AQol-8D.

##### Other Measures

Depression symptom severity will also be rated by a clinician (clinical psychologist or clinical psychologist in training), using a short version of the Hamilton Rating Scale for Depression (HAM-D) with 6 items. The chosen version best differentiates between groups with and without treatment compared with other versions [62]. HAM-D is the most widely used clinician-administered depression assessment scale. The rating scale was supplemented with the Structured Interview Guide for the HAM-D (SIGH-D) [63,64]).

The therapeutic alliance is measured with a shortened version of the Working Alliance Inventory (WAI-SR) [65] is used, which contains the 8 items of the dimensions (process and goals).

Negative intervention effects were assessed with the Inventory for Balanced Assessment of Negative Effects of Online Interventions (INEP-ON) [66], which captures negative intervention effects from the patient’s perspective. The questionnaire also assesses whether patients attribute these effects to the intervention or to other factors.

##### Confounding Variables

To estimate the influence of potential confounding variables, additional information is collected (see Textbox 1).

**Confounding variables to be investigated in secondary analyses.**

**Moderators (to be recorded before randomization)**

- Duration of illness
- Gender and age
- Psychiatric comorbidity
- Type of depression (first-time, recurrent)
- Severity of depression (Patient Health Questionnaire-9 sum score at randomization)
- Prior treatment (antidepressant medication, outpatient psychotherapy, inpatient treatment or day treatment for a mental disorder, and comparable digital interventions for depression based on cognitive behavioral therapy)
- Personality functioning
- Health literacy

**Mediators (to be recorded after 8 weeks and after 6 months, respectively)**

- Outpatient visits with psychotherapists or psychiatrists
- Outpatient visits with general practitioners
- Outpatient visits with physicians of other specialties
- Inpatient treatment for mental disorders
- Changes in medication use between baseline and follow-up
- Use of other digital interventions addressing mental health between baseline and follow-up
- Use of alternative treatment methods (eg, acupuncture, osteopathy, homeopathy, and Reiki) between baseline and follow-up
- Use of low-threshold psychosocial services (eg, self-help groups, counseling centers, and social psychiatric service) or preventive services (eg, relaxation training) between baseline and follow-up
- Critical life events between baseline and follow-up

Personality functioning is assessed with the Level of Personality Functioning Scale-Brief Form (LPFS-BF) [67], a brief self-report measure that captures personality functioning in the domains of self and interpersonal pathology and is compatible with the assessment of severity of personality dysfunction according to ICD-11. The German version has high reliability and satisfactory validity. The severity of personality dysfunction was an important predictor of trial discontinuation in a previous study [50].

Health literacy was assessed with the Mental Health Literacy Questionnaire (MHLq) [68], a 29-item scale with 4 dimensions (knowledge about mental health problems, false beliefs or stereotypes, help-seeking and first aid skills, and self-help strategies). Results showed significant differences between individuals with more or less experience with mental health and good internal consistency for the overall outcome.

### Safety End Points

To assess the safety of the intervention, the following adverse events were defined and recorded at 8 weeks and at 6 months, respectively: (1) new occurrence or worsening of suicidal ideations during the intervention and follow-up period (recorded via item 9 of the PHQ-9), (2) illnesses and injuries during the intervention and follow-up period (self-report), and (3) inpatient treatment during the intervention and follow-up period (self-report).

### Sample Size

The primary end point is the between-group difference in depressive symptoms (PHQ-9 total score) after 8 weeks. The effect regarding the primary end point will be determined by a 2-tailed *t* test. Based on the results of our previous study, an effect of *d*=0.4 (Cohen *d*) is expected and a dropout rate of up to 60% is assumed. A sample size of 570 participants is required to detect the expected difference at 85% probability and α=5% significance in both intention-to-treat and per-protocol analyses.

### Data Management

#### Study Data

All study data are collected and stored in an electronic data capture system (Climedo) in the form of electronic case report forms and electronic patient reported outcome forms All data collected by means of the EDC provided by Climedo Health GmbH are processed and stored in data centers in Frankfurt am Main, operated by Amazon Web Services EMEA SARL (Luxembourg), which acts as an order processor for Climedo Health GmbH. Amazon Web Services is currently ISO 27001, ISO 27017, and ISO 27018 certified hosting provider. The ISO 27001 certification largely corresponds to the requirements of the German Federal Office for Information Security and is compatible with these (eg, BSI-200). ISO 27017 is an international standard for securing cloud services (Cloud Security), while ISO 27018 primarily relates to the protection of personal data (Cloud Privacy). All personal data are stored on servers in Germany. After completion of the data collection, the pseudonymized data will be exported by MindDoc Health GmbH and stored there in digital form for a period of 10 years in accordance with legal requirements.

All data collected from participants will be assigned an 8-digit code consisting of random numbers and letters. Personal information, including name and contact details, are only collected from those participants who do not meet any exclusion criteria in the screening.

A pseudonymization list of names assigned to codes is stored in a protected area of the electronic data capture system, only accessible to authorized members of the study team. The data are protected from unauthorized access, allowing for secure study procedures. The list will be destroyed after the study completion or after 10 years.

All data collected will be handled in accordance with national law and the GDPR. All personal and health data will be stored and handled in accordance with international and national data protection guidelines. Any publication derived from the personal data will be presented in such a way that it is impossible to identify individuals by referring only to group-level statistics. Participants have the right to request the deletion of their records prior to the destruction of the pseudonymization list without giving any reason.

#### App Data

To use the digital health app, participants must create a user account with a name and email address of their choice, stored separately by MindDoc Health GmbH. Passwords must meet minimum security standards that are checked in real time by an algorithm. Forgotten passwords cannot be recovered and a new password can be set through a link sent via email.

Using the app generates additional usage data (eg, answers, mood ratings, and course or exercise usage) stored by MindDoc Health GmbH. These data can be merged with study data in pseudonymized form using personalized access codes and a 16-digit user ID.

All data collected as part of the app are processed and stored in a high-security data center in Germany. The data center is operated by T-Systems International GmbH, Hahnstraße 43, 60528 Frankfurt am Main, Germany, which acts as an order data processor for MindDoc Health GmbH. All personal data are stored on servers in Germany. Separate consent is obtained for the processing of health data pursuant to Article 9 (2) lit h DS-GVO within the app, without which the app cannot be used. Irrespective of the right to data deletion pursuant to Art. 17 DS-GVO, the data collected in the app can be deleted from the server directly in the app in the “Settings → Data & Security” area at any time. Prior to this, they can be transferred to the user’s own device using an export function.

All communication between the user and the technical platform is automatically encrypted. The app communicates with the server over encrypted connections using transport layer security or hypertext transfer protocol secure, which prevents third parties from unauthorized reading of the data while it is being sent (ie, “in transit”). Both servers and databases are located behind firewalls to prevent access to data while it is being stored (ie, “at-rest”) or while it is being processed (ie, “in process”). In order to independently check system security, test attacks (penetration tests) are carried out at least once a year by an externally contracted IT security company on the servers provided and attempts are made to gain unauthorized access to them on a test basis.

### Statistical Analysis

Data will be described using descriptive statistics (eg, frequencies, mean, median, SD, and IQR) and visualizations (eg, histograms, box plots, and bar charts). Assumptions for statistical tests will be checked (eg, normality by histograms and tests, sphericity by Mauchly test, and equal variance-covariance matrices by Box and Levene tests). App usage will be described according to previously proposed standards for reporting adherence to digital interventions [69], and include days of use and engagement with different features of the app.

Significant baseline group differences will be detected using *χ*^2^ and *t* tests on baseline variables and accounted for in all analyses.

The intervention’s effectiveness will be analyzed using an intention-to-treat approach with multiple imputed data. Missing values from the postsurvey and follow-up survey of nonbinary end points will be imputed using all available information collected at baseline by applying an iterative Markov chain Monte Carlo method based on the initial group assignment. For this purpose, imputations will be calculated for 100, 150, 200, 300, 400, and 500 iterations. Then, the fraction of missing information (FMI) index will be determined for all multiply imputed data sets. The FMI ranges from 0 to 1 (where 1 means that 100% of the information required for the planned conclusions is missing) and is a reliable indicator of the validity of conclusions based on imputed data sets [70]. For the following analyses, the number of imputations that do not result in further improvement in FMI compared to previous imputations will be chosen. Statistical characteristics will be aggregated across the multiply imputed data sets using Rubin’s rules. The comparison of intervention effects on all outcomes will be done using *t* tests.

In addition to the primary analysis, further sensitivity analyses will be performed. Analyses of intervention effects on all outcomes will be repeated using analysis of covariance, adjusted for baseline scores. In addition, per-protocol analyses excluding participants who violated the protocol will be conducted. The following protocol violations have been prespecified: (1) failure to download the app and complete the onboarding process in the intervention group, (2) reporting of using a similar digital health intervention during the intervention and follow-up period in the control group, (3) reporting of regular psychotherapy during the 8-week intervention period, and (4) noncompletion of postintervention or follow-up assessment. Subgroup analyses will be done to address potential heterogeneity.

To allow for adjustment of the sample size in this study (for lower dropout), an interim analysis can be performed. This can be done once the primary end point of 182 participants is available. With this sample size, the expected effect can be revealed with a probability of 85% at a significance level of α=10%. The interim analysis will be performed blinded, meaning that the variable “group membership” will be replaced by a dummy variable and the blinded data set will be analyzed by a person who is not part of the core study team and is unaware of the group assignment.

### Ethical Considerations

This trial is conducted in compliance with the protocol, the Declaration of Helsinki and good clinical practice. The local ethical committee of Ludwigs-Maximilians-Universität München has approved the protocol (project 22-0984). Amendments to the trial protocol will be immediately communicated to the local ethical committee as well as the trial registry by the corresponding author.

No anticipated risks beyond normal daily life are associated with using the app and participating in the study. Temporary mood changes or increased stress may occur during mental health treatment. Based on preliminary studies with the DiGA, a medical benefit, including a reduction of depressive symptoms, can be assumed.

Participants will not receive compensation for participating in the study, but both groups will get free access to the intervention for a year, and those who complete assessments are eligible for a monthly prize draw for a €50 (US $52) voucher. All measures used in the study were selected both based on psychometric properties and the number of items in an effort to minimize participant burden.

The participants’ physical, psychological, and social integrity will be protected. The participants have the option to refuse to answer questions, end participation at any time, and request data deletion (before their data have been anonymized) without any consequences.

If suicidal tendencies are suspected or present before randomization, applicants will not be eligible to participate in the study. If suicidal ideations are reported during the web-based screening process, participants will receive written information on available help resources. If suicidal ideations are detected during the diagnostic interview, the interviewer will conduct a crisis intervention and refer the applicant to the local crisis service.

Since suicidal ideations are among the symptoms of depression, they may occur during the intervention or follow-up period even in participants who did not report them at baseline. In this case, precautions will be taken: (1) participants will receive written information about help resources immediately after completing assessments and (2) the study team will be informed and a member will promptly contact participants to further assess the situation. If a crisis cannot be resolved or a participant cannot be reached, support from local emergency services will be sought and the participant’s contact information will be shared with these services. Participants agree to this procedure upon enrollment in the study.

### Patient and Public Involvement

Patients or the public were not involved in the design, conduct, reporting, or dissemination plans of our research, although user or patient feedback was an important source for the development and improvement of the mental health app investigated in this trial.

### Dissemination

This trial aims to support the eligibility of MindDoc with Prescription for registration as a DiGA (Digitale Gesundheitsanwendung) for patients with mild to moderate depression under the Digitales Versorgungsgesetz (Digital Health Care Act). This law regulates the cost reimbursement by German statutory health insurances for digital health applications.

The publication plan includes a report on the effects of MindDoc with Prescription on outcomes, and a second publication on predictors of effectiveness in low-threshold mental health interventions. Results will be presented at conferences and authorship will be based on contributions.

## Results

Recruitment for the trial started on February 7, 2023, and the first participant was randomized on February 14, 2023. As of September 5, 2023, a total of 275 participants have been included in the trial and 176 have provided the primary outcome. The rate of missing values in the primary outcome is approximately 20%.

## Discussion

### Anticipated Findings

Data from this study will be used to establish whether access to an app-based, self-guided, transdiagnostic intervention addressing common mental disorders is associated with an improvement of depression symptoms in individuals diagnosed with mild or moderate depression and with or without comorbid mental disorders. It will also be used to investigate improvements in quality of life, anxiety symptoms, and patient empowerment and self-management behaviors in relation to access to the intervention.

The results will provide additional insights into the therapeutic alliance in digital interventions and also assess their safety by systematically collecting data on negative intervention effects.

The study will contribute to the evidence base on transdiagnostic digital interventions and at the same time provide information on what role primary care providers can play in facilitating the dissemination of digital mental interventions.

### Limitations

A possible limitation of the study could be the composition of the sample. Since participants are recruited over various channels including press releases and social media advertising, it is possible that those with previous (positive) experience with psychotherapy or other psychological interventions are more likely to enroll than those who have not yet sought treatment. In our previous trial, almost 60% of the participants reported previous inpatient or outpatient treatment for a mental disorder. In this trial, we aim to mitigate the risk of an imbalanced sample by cooperating with primary care providers (general practitioners and family doctors) to recruit participants for the trial.

Another possible limitation could be a high dropout rate at postintervention or follow-up, as is common in self-guided interventions. While we determined the sample size accounting for that risk, we also implemented several mitigation measures to reduce dropout, including clinical telephone interviews at baseline and postintervention.

The study is conducted in Germany. Results may not be generalizable to other countries and health care systems, especially free-market health care systems.

### Impact and Future Directions

With a focus on both symptom reduction and patient empowerment and self-management behaviors, the study will contribute to expanding the evidence base of transdiagnostic low-threshold digital interventions.

Furthermore, the study aims to provide insights into factors impacting the effectiveness of transdiagnostic digital interventions in mental health care. By examining potential moderators and predictors of outcomes, the study will inform decision-making in stepped care and improve mental health care.

General psychopathology and personality dysfunction have previously been shown to predict treatment response in digital interventions, rendering these factors as potential predictors of outcome in this trial. Further, improvement of mental health–related self-management skills and literacy through app usage may constitute an important mediating factor for change in psychopathology. In addition, the detailed assessment of help-seeking behavior and mental health care use may help to disentangle the direct and indirect effects of transdiagnostic digital interventions on mental health.

## Appendix

Multimedia Appendix 1 Description of MindDoc with Prescription.

## Abbreviations

AQoL-8D: Assessment of Quality of Life-8 Dimensions
AUDIT: Alcohol Use Disorders Identification Test
DiGA: Digital Health Application
DSM-5: Diagnostic and Statistical Manual of Mental Disorders, Fifth Edition
DUDIT: Drug Use Disorders Identification Test
EDC: electronic data capture system
FMI: fraction of missing information
GAD-7: Generalized Anxiety Disorder-7
GDPR: General Data Protection Regulation
HAM-D: Hamilton Rating Scale for Depression
ICD-10: International Classification of Diseases, 10th Revision
INEP-ON: Inventory for Balanced Assessment of Negative Effects of Online Interventions
LPFS-BF: Level of Personality Functioning Scale-Brief Form
MHLq: Mental Health Literacy Questionnaire
PHQ-9: Patient Health Questionnaire-9
PRIME-MD: Primary Care Evaluation of Mental Disorders
SIGH-D: Structured Interview Guide for the Hamilton Rating Scale for Depression
WAI-SR: shortened version of the Working Alliance Inventory

## Data Availability

The data sets generated and analyzed during this study will be available from the corresponding author on reasonable request after the main results of the trial have been published in a peer-reviewed journal.
