# Supplementary material for: Improving Mild to Moderate Depression With an App-Based Self-Guided Intervention: Protocol for a Randomized Controlled Trial
Source: JMIR Res Protoc. 2023 Oct 25;12:e46651. doi: 10.2196/46651 (PMC10632923; doi:10.2196/46651)
Supplement: Multimedia Appendix 1 [file resprot_v12i1e46651_app1.pdf]

Description of “MindDoc with Prescription”

Intended Medical Purpose

"MindDoc with Prescription" allows users to log signs and symptoms of common mental illnesses in real time over long periods of time.

- The application enables users to self-manage symptoms and related problems by providing evidence-based transdiagnostic courses and exercises to help identify, understand, and manage symptoms through self-initiated behavior change.
- The application provides users with regular guidance on whether further medical or psychotherapeutic evaluation is indicated through a general feedback on emotional health.

"MindDoc with Prescription" explicitly does not replace a medical or psychotherapeutic assessment or treatment, but can prepare and support the path to psychiatric or psychotherapeutic treatment.

Mode of Operation

The application provides an assessment tool which allows users to monitor signs and symptoms of common mental health problems and related problem areas in real-time over long time periods. Questions are asked within three blocks a day (morning, noon, evening), with each block consisting of three or more questions. In contrast to a static mood-diary or the repeated completion of static questionnaires, the underlying algorithm adjusts the number and area of questions to the answers of the user as well as to the completion rate of previous questions blocks. In addition, every question block is finished by a general mood-tracking (very bad, bad, moderate, good, very good) as well as the opportunity to track emotions and situations via text entry, pre-defined or customized tags (positive, negative, neutral).

This information is then processed to continuously provide individualized automated feedback (insights) to the user that reflect symptoms as well as potential triggers and problem areas and suggest suitable disorder-specific and trans-diagnostic self-management courses and exercises to address symptoms and problems.

As soon as the user has answered the required minimum of questions, the application provides an individualized medical orientation regarding the need for assessment of their mental-mental condition which can be shared with a specialist.

The application has four interconnected core components.

1. Continuous monitoring of symptoms of common mental disorders, related problems, and personal resources (Journal).

2. Automated feedback on general symptom load and level of functioning (Results)

3. Automated feedback on symptoms, symptom clusters and relevant problem areas, along with recommendations for exercises and courses (Insights)

4. Structured self-management courses and exercises that address problems that commonly contribute to mental health disorders (Self-Management)

Core components

Journal

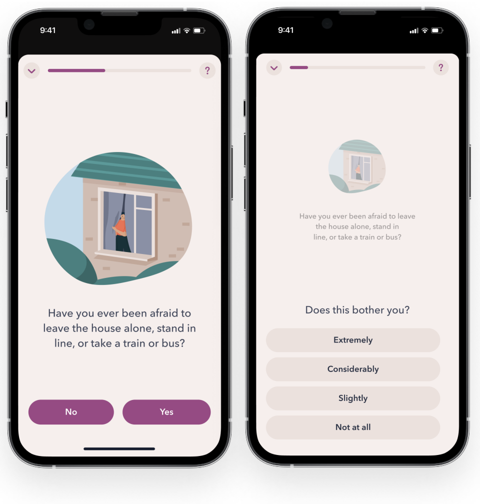

Users answer up to three blocks of questions per day on symptoms of common mental disorders, related problems and personal resources that can be helpful to address those symptoms and problems. Questions are chosen from a large question pool based on an adaptive algorithm that takes into account previous answers of the user. Thus, the more questions are answered, the more individualized and relevant the questions become.

Questions typically require a dichotomous answer. If a symptom is confirmed, a follow-up question is asked to assess symptom severity (“How much does this bother you?), which is rated on a four-point scale ranging from 1 to 4 with a visual anchor.

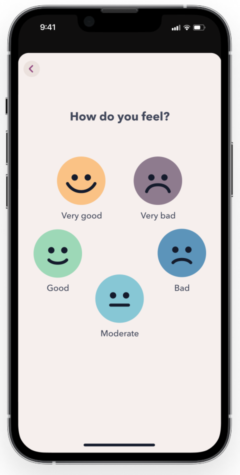

At the end of each question block, users can rate their current mood on a simple 5-point scale (very bad, bad, neutral, good, very good). Users can also add personal notes as well as predefined and customized tags to their entries. The mood rating and the notes can be used independently of the question blocks, so users can make multiple entries per day.

A customizable alert function can be set to remind users to answer the question block at respective times via push notifications for higher response rates.

Results

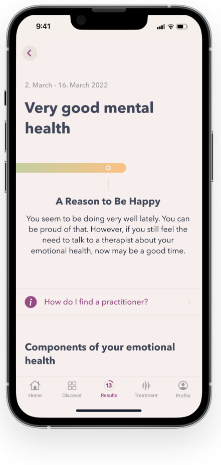

If users have answered a minimum number of questions within a period of 14 days, they will get feedback on their answers regarding their symptom load. Users with a high symptom load, a high level of functional impairment or critical symptoms like suicidal tendencies are recommended to seek further assessment.

In addition to that recommendation, users are provided with summaries of their answers over predefined periods of time as well as weekly, monthly and yearly statistics. Some summaries can be exported in PDF format and can be shared with treatment providers.

Users who report suicidal tendencies are instantly and automatically directed to an automated dialogue (crisis-bot) that culminates in a direct connection to a local crisis hotline if the user agrees. In Germany, this is the Telefonseelsorge, in other countries it is a comparable service. Alternatively, the user is guided to contact a friend or family member via text message to seek help.

Insights

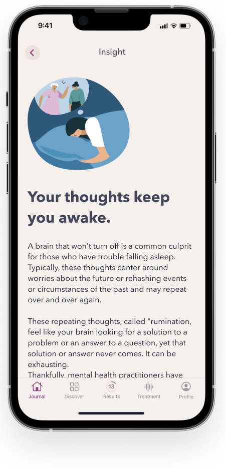

If users report specific symptoms or problems, or combinations of symptoms and problems repeatedly, they will receive automated feedback via insights.

Insights provide general information about symptoms, about behavior-health-links or about consequences of behaviors. They also provide an overview of possible strategies to address problems and prompt further engagement with related self-management exercises.

Self-management

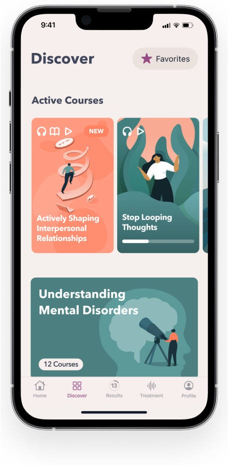

The application provides a library of courses and exercises that are designed to enable the user to self-manage mental health problems. Courses can be accessed at the discretion of the user, and subscribed (i.e., full access) users have full access to the entire course library.

Courses consist of multiple exercises. For some courses it is advisable to complete the exercises in the set order, or to allow for time to implement behaviour changes between two exercises. If this is the case, this is explained to the user within the exercise.

The version of the application that has been evaluated contained the content described in the table below.

**Course**	**Domain**	**Learning goals**
Depression	Psychoeducation	Knowledge about signs and symptoms, prevalence, etiology, treatment options.
Social anxiety disorder	Psychoeducation	Knowledge about signs and symptoms, prevalence, etiology, treatment options.
Generalized anxiety disorder	Psychoeducation	Knowledge about signs and symptoms, prevalence, etiology, treatment options.
Specific phobias	Psychoeducation	Knowledge about signs and symptoms, prevalence, etiology, treatment options.
Panic disorder and agoraphobia	Psychoeducation	Knowledge about signs and symptoms, prevalence, etiology, treatment options.
Health anxiety	Psychoeducation	Knowledge about signs and symptoms, prevalence, etiology, treatment options.
Chronic pain	Psychoeducation	Knowledge about signs and symptoms, prevalence, etiology, treatment options.
Eating disorders	Psychoeducation	Knowledge about signs and symptoms, prevalence, etiology, treatment options.
Insomnia	Psychoeducation	Knowledge about signs and symptoms, prevalence, etiology, treatment options.
Pre- and postnatal disorders	Psychoeducation	Knowledge about signs and symptoms, prevalence, etiology, treatment options.
Outpatient Psychotherapie	Psychoeducation	Knowledge about different types of psychotherapy and how to get treatment
Inpatient treatment	Psychoeducation	Knowledge about different options of inpatient treatment (e.g., psychiatric, psychosomatic, rehabilitation) and indications
Entering the World of Mindfulness	Self	Knowledge about the concept of mindfulness and meditation, practice mindfulness
Jump-starting Mindfulness in Seven Days	Self	Knowledge about the concept of mindfulness and meditation, practice mindfulness
Learn Self-Kindness for greater Happiness	Self	Learn about the role and practice self-compassion, strengthen self-esteem, cognitive restructuring of core beliefs about the self.

Thoughts and Beliefs	Thinking	Identification of automatic thoughts, thinking styles, cognitive distortions, application of techniques of cognitive restructuring
Stop Looping thoughts	Thinking	Knowledge about rumination and strategies to interrupt or cope with it, application of those strategies
Thoughts about suicide	Thinking	Knowledge about suicidal ideations, instructions to set up an emergency plan, and encouragement to seek professional help
What am I feeling?	Feeling	Knowledge about the role of emotions, the interrelation of emotions and needs, accept unpleasant emotions
Why am I feeling this way?	Feeling	Knowledge about the interrelation between thoughts, emotions and actions, identify needs behind emotions, accept unpleasant emotions
Get active	Take Action	Behavioral Activation
Facing your fears	Take Action	Understanding the rationale of exposition, role of avoidance interoceptive exposition, graded in-vivo exposition,
Dealing with Challenges and Problems	Take Action	Problem solving<br>Radical Acceptance
Actively Shaping interpersonal Relationships	Relationships	Improve social skills, achieving interpersonal goals
Dealing with Interpersonal Conflicts	Relationships	Improve social skills, handle conflicts
Progressive Muscle Relaxation	Relaxation	Practice progressive muscle relaxation
Autogenic training	Relaxation	Practice Autogenic Training
Improve your Sleep	Body	Knowledge about sleep architecture, sleep cycles, guidance to establish sleep hygiene, instruction for sleep compression
Intuitive Eating	Body	Knowledge about intuitive eating, guidance to establish a balanced diet
Exercise and Mental Health	Body	Knowledge about the benefits of exercise on mental health, guidance to establish a regular exercise schedule
